# New modalities for non-invasive positive pressure ventilation: A review article

**DOI:** 10.22088/cjim.10.1.1

**Published:** 2019

**Authors:** Shahram Seyfi, Parviz Amri, Simin Mouodi

**Affiliations:** 1 Clinical Research Development Unit of Ayatollah Rouhani Hospital, Babol University of Medical Sciences, Babol, Iran; 2 Cancer Research Center, Health Research Institute, Babol University of Medical Sciences, Babol, Iran; 3 Social Determinants of Health Research Center, Health Research Institute, Babol University of Medical Sciences, Babal, Iran

**Keywords:** Non-invasive ventilation, adaptive servo-ventilation, acute respiratory failure

## Abstract

Efficiency of non-invasive positive pressure ventilation in the treatment of respiratory failure has been shown in many published studies. In this review article, we introduced new modalities of non-invasive ventilation (NIV), clinical settings in which NIV can be used and a practical summary of the latest official guidelines published by the European Respiratory Clinical Practice. Clinical trials and review articles in four databases up to 25 February 2018 about new modalities of non-invasive positive pressure ventilation were reviewed. Commonly used modalities for treatment of respiratory failure include: CPAP (continuous positive airway pressure) and BiPAP (bilevel positive airway pressure) or NIPSV (noninvasive pressure support ventilation). The limitations of the BiPAP method are the trigger and cycle asynchrony, inadequate volume delivery and increased respiratory rate. Newer methods, such as adaptive servo-ventilation, have been developed to treat central and complex sleep apnea and the NAVA (neutrally adjusted ventilatory assist) to improve the trigger and cycle asynchrony.

In the proportional assist ventilation, unlike the pressure support ventilation, with increased patient effort (flow) the tidal volume increases and it prevents the increase in the respiratory rate and respiratory distress. High-flow nasal cannula is a non-invasive technique that does not provide respiratory support, but provides a mixture of oxygen to the patient. The use of non-invasive pursed-lip breathing ventilation in chronic obstructive pulmonary disease (COPD) patients reduces dyspnea (decreases respiratory rate) and increases blood oxygen saturation. New modalities of NIV improve patient comfort and patient–ventilator interactions, and are recommended in patients with respiratory failure.

The main cause of the mechanical ventilation is acute respiratory failure (1). Mechanical ventilation is performed in two ways, invasive and non-invasive ventilation. The mechanical ventilation of the lungs using mask is called non-invasive positive pressure ventilation (NIPPV) (2, 3). Evidence suggests that non-invasive ventilation compared to invasive positive pressure ventilation (IPPV) decreases the risk of ventilator associated pneumonia (VAP), sinusitis, sepsis, and ultimately reduces the rate of hospitalization and mortality (4). The present study aimed to represent the new modalities for non-invasive positive pressure ventilation and their clinical indications. 

## Methods

**Data sources and search criteria: **We searched clinical trials and review articles in four databases (Pubmed, the Cochrane Database of Systematic Reviews, Scopus and Magiran) up to February 25, 2018.

At first, all clinical trials, systematic reviews and review articles which used the entry terms "positive end-expiratory pressure" OR "positive-pressure ventilation" OR "positive-pressure respiration" in title, abstract or keywords were included. Subsequently, the manuscripts that mentioned non-invasive positive pressure ventilation and emphasized on new modalities of this procedure were selected. We also reviewed the reference lists of included studies to identify review articles related to search criteria. As well, we only searched Persian and English language published manuscripts. Two reviewers updated the abstract search. Ethics committee approval was not required for this review article.

**Study selection: **We included clinical trials and review articles that reported indications and advantages of new modalities of NIPPV. 

**Data extraction and quality assessment: **Two authors, unblinded to data sources, summarized the included studies. Quality assessment was performed with emphasis on reporting a new modality on application of NIPPV and outcome reporting. 

## Discussion

We included 16 clinical trials, review articles or systematic reviews which used the mentioned keywords in the title, abstract or keywords and emphasized on NIPPV new modalities and their clinical indications.

**Indications for non-invasive ventilation: **Non-invasive ventilation indications are extensively increasing. One of the common indications of NIPPV is the COPD treatment, as well as the weaning of these patients from invasive ventilation (5, 6).

Other non-invasive ventilation indications include: Asthma, upper airway obstruction, mild to moderate hypoxemia, central apnea or hypoventilation syndrome (Table 1). The exclusion criteria for NIPPV included: Immediate cardiac arrest, hemodynamic instability, shock, life threatening cardiac ischemia, dangerous arrhythmias, patients who do not cooperate, increased discharge and airway surgery.

In cases of acute and short-term diseases, an oronasal mask, and in chronic and prolonged cases, a nasal mask should be used; and if the patient does not cooperate well, full face masks are recommended (4, 6, 7, 9). In NIPPV, all ventilation techniques are applicable, but CPAP (continuous positive airway pressure) and BiPAP (bilevel positive airway pressure) or NIPSV (noninvasive pressure support ventilation) + PEEP (positive end-expiratory pressure) are the most commonly used methods (4, 6, 7, 9).

**Table 1:** **Indications for non-invasive positive pressure ventilation** **(** 6 **-** 8 **)**

**Strong evidence (Level A)** Acute exacerbations of COPD To facilitate weaning in COPD Acute cardiogenic pulmonary edema (use of CPAP) Immunocompromised patients **Reasonable evidence (Level B)** Postoperative respiratory failure Asthma Not intubating patients Obstructive sleep apnea or obesity hypoventilation **Cohort series, case reports (Level C)** During bronchoscopy Cystic fibrosis Restrictive disorders Upper airway obstruction Acute respiratory distress syndrome

COPD: chronic obstructive pulmonary disease CPAP: continuous positive airway pressure

**NIPPV setting: **CPAP is used in patients with acute congestive heart failure and obstructive sleep apnea (OSA). In patients with obstructive sleep apnea, the CPAP is adjusted between 5 to 12 centimeters of water during sleep, after attaching the mask.

BiPAP is the most commonly used mode in noninvasive method. In patients with COPD and other patients, after attaching the mask on the patient's face, the setting will be 3-5 cm of water expiratory positive airway pressure (EPAP) and 5-15 cm of water inspiratory positive airway pressure (IPAP). 

EPAP is initially set on lower number, and like PEEP in case of hypoxemia, it can be set on the maximum of 10-12 cm/H2O. 

**Figure1:**
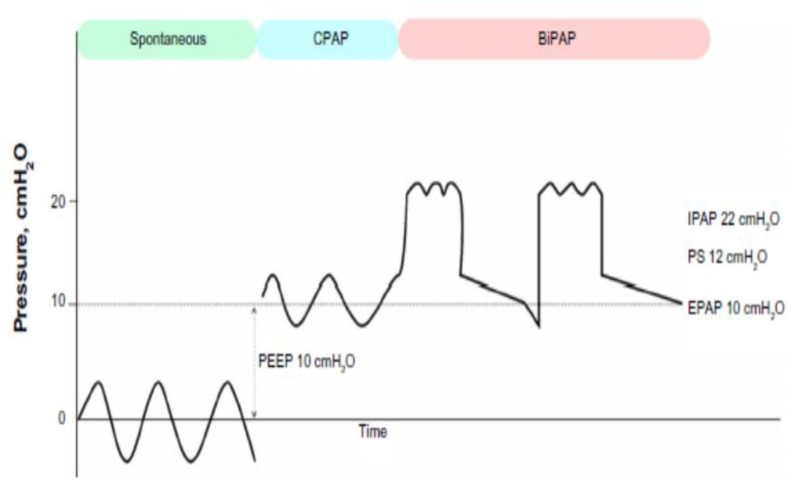
Pressure/time curves in spontaneous, continuous positive airway pressure (CPAP) and BiPAP, Pressure support (PS). Positive end expiratory pressure (PEEP), Inspiratory positive airway pressure (IPAP), expiratory positive airway pressure (EPAP) (12).

If the patient has hypercarbia or tachypnea, the IPAP increases to a maximum of 20-25cm/H2O. The difference between the designated IPAP and the EPAP (figure 1) is called PS (5, 8-14) 

Blood pressure, heart rate, respiratory rate, Paco2, PH and PaO2 should be monitored during NIPPV. When non-invasive ventilation is effective, the above mentioned variables tend to be normal. The application rate of NIPPV depends on the severity of the underlying disease and the patient's admission and varies from one hour to 23 hours a day (4, 6, 9). The limitations of the CPAP method include the complications related to the mask situation on the face such as wound and necrosis of the nasal bridge resulted in patients’ speaking and eating (4, 6, 9) .The limitations of the BiPAP procedure include the complications of mask intolerance, eye damage, eating and talking and phlegm disorder, patient-ventilator asynchrony including trigger and cycle asynchrony (15).

**PAV (Proportional assist ventilation): **This PSV-like mode is a spontaneous breathing support mode. In fact, it is pressure controlled, patient triggered, pressure limited and flow cycled; however, in the case of hypercarbia, the ventilator feels the patient's increased sensitivity to the inhale flow, and, in contrast to the PS, it increases the current volume instead of increasing the number of respiration; therefore, it imitates the natural respiration pattern which COPD patients can tolerate much better. In this mode, operator sets the percentage of the assistance for the current volume, the percentage of flow assistance (based on resistance and elastance). PEEP and FIO2 should be adjusted. In this mode, the risk of Auto-PEEP and the disproportionate support level will be reduced (6, 16, 17). This mode is indicated in patients who had asynchrony with NIPSV. The advantages of this mode include better compatibility than NIPSV and prevention of over-assistance (18).

**Adaptive servo ventilation (ASV): **A large proportion of patients with chronic heart failure may have chyne stoke respiration. In addition, some of them have coincidently OSA and CSA. An increase in CO2 can exacerbate heart failure. In these cases, CPAP alone cannot help. In the ASV mode, which was initially used to treat central apnea, it is possible to prevent obstructive apnea by regulating exhaust-pressure during expiration. In this mode, the inspiratory pressure and PEEP are changing based on the patient's respiratory pattern. The use of ASV can be dangerous in CHF patients with sleep disorders and low EF (17-20).

**NAVA (Neurally adjusted ventilator assist): **In the NAVA, the ventilator detects the contraction signal of the aperture by the EMG electrode located in the lower esophagus, and controls the ventilator, flow, volume and pressure without delay. Unlike the PAV, the patient's respiratory center controls all auxiliary breathing process from the trigger to the end of the inspiratory cycle. Currently, this mode has the highest degree of coordination in starting ventilation and maximum compatibility with the patient’s inspiration. Its limitation includes the necessity for esophageal catheter device (7, 18, 21, 22).

**AVAPS (Average volume assured pressure support) ventilation: ** AVAPS is one of the intelligent ventilation modes that delivers the preset volume securely by changing the pressure support level. Studies have not shown the superiority of this mode in comparison with NIV in the conventional way. Its limitation includes increased inspiratory pressure in cases of low lung compliance. Its main indication was observed in COPD patients and hypoventilation syndrome (18, 23, 24).

**HFNC (High flow nasal cannula): **HFNC is a new method for oxygen therapy that provides warm and humid oxygen to patients with special tools using the flow up to 60 liters per minute with FIO2=21- 100% (9, 25, 26). HFNC creates a low level of PEEP, reduces airway resistance, and washouts air chips. The benefits of HFNC are the lack of a ventilator, good compliance with the patient, and improve oxygenation. Its main disadvantage is the lack of ventilation support. The main indications of its application are hypoxemic respiratory failure, acute heart failure requiring a long NIV, and mechanical ventilation weaning (18). Treatment of underlying diseases and oxygen are the basis of the AHRF treatment. Oxygen is prescribed in nasal cannulas, or face mask, NIV, and MV. 

The conventional oxygen rate given by the face mask or nasal cannula methods is the maximum of 15 liters per minute, which is not enough to provide oxygen for the hypoxic patient (27).

Yue-nan et al. compared the effects of HFNC with humid oxygen and NIPPV in the treatment of acute respiratory failure in a review article. Eighteen papers and 3881 patients were examined for ICU tracheal intubation and mortality. There was no significant difference between HFNC and NIPPV; however, the amount of intubation was lower comparing to conventional HFNC oxygen therapy. In terms of mortality and duration of hospitalization in ICU, HFNC showed no superiority on conventional oxygen therapy and NIPPV (27, 28). Okuda et al. examined the effect of HFNC on the parameters of respiratory physiological parameters through esophageal balloon in 10 patients, with off, 30 L / min and 50 L / min methods. The volume of flow and PEEP increased with increased flow and decreased respiratory rate (20, 29).

**PLB (Pursed lips ventilation): **PLB is a strategy in which the air exits slowly and is controlled by puckered lips during exhalation. PLB has indication for COPD patients who have dyspnea. PLB improves ventilation, slows retention of air, prolongs airway opening, reduces respiratory rate, reduces respiratory rate, outflows of old air and brings the new air flow to the lungs. In this method, the patient sits on the chair, relaxes the shoulder and neck muscles; then slowly inhales with the nose for 2 seconds, and exhales slowly with puckered lips within 4 seconds (30, 31).

**NIHFV (Non-invasive high-frequency ventilation): **NIHFV is a relatively new mode which is more commonly used in non-invasive ventilation for infants. In this mode, the flow is less than the anatomical dead space (2ml / kg) with high respiration rate (80- 1600 per minute or 3-10 Hz) and mean airway pressure (MAP) (29, 32).

**Recommendations for using NIV for the treatment of acute respiratory failure based on the official ERS / ATS clinical practice guidelines: (** 4 **,** 7 **,** 9 **)**

- Bi-level NIV is a selective method in treatment of COPD exacerbation as well as preventing intubation in these patients.
- Bi-level NIV or CPAP is recommended in patients with cardiogenic pulmonary edema.
- There is insufficient evidence to suggest the use of NIV in asthmatic patients.
- Early NIV is recommended for the treatment of acute respiratory failure in immunocompromised patients.
- There is insufficient evidence to suggest the use of NIV in patients with NOVO ARF.
- NIV is recommended for post-operative ARF.
- NIV is suggested for end stage cancer patients with dyspnea.
- NIV is recommended for the treatment of acute respiratory failure after chest trauma.
- There is insufficient evidence to suggest using NIV in patients with pandemic viruses.
- NIV is recommended for prevention of respiratory failure after extubation in high risk patients. This method should not be used in the treatment of respiratory distress after extubation.
- NIV is recommended for the isolation of ventilators in patients with hypercapnic respiratory failure, but there is not enough evidence to support the treatment of hypoxic respiratory failure.

The advantage of our study is to describe the indications and clinical aspects of the most important new modalities of NIPPV. A few clinical trials or review articles have been concerned on technical approaches of all new modalities of NIPPV, in recent years. Some studies described a number of these modalities according to target patients; for example, Masip described the indications and clinical considerations of CPAP and NIPSV in acute heart failure patients (22). Elgebaly compared intermittent positive pressure ventilation (IPPV) and NIPPV in the management of acute respiratory failure and hemodynamic parameters after open-heart surgery (15) and Ferreira evaluated NAVA and PSV in critically ill patients (24). 

In our study, we described all these new modalities which can be used in the conditions which sufficient human resources and equipment are not available, especially in developing countries.

It is recommended for future researches to design and perform randomized clinical trials to compare the impacts of all of these new modalities in the different aspects of patients’ treatment. Furthermore, the implementation of a new modality of NIPPV –as an intelligent system- which can trigger the patient’s respiration depending on end-tidal carbon dioxide concentration and regulate the respiratory rate, automatically is suggested. 
